# Ordered Hierarchical Porous Structure of PtSn/3DOMM-Al_2_O_3_ Catalyst for Promoting Propane Non-Oxidative Dehydrogenation

**DOI:** 10.3390/nano13040728

**Published:** 2023-02-14

**Authors:** Yuanqing Sun, Bohan Feng, Qian Lian, Chengshu Xie, Jing Xiong, Weiyu Song, Jian Liu, Yuechang Wei

**Affiliations:** 1 State Key Laboratory of Heavy Oil Processing, College of Science, China University of Petroleum, Beijing 102249, China; 2 Key Laboratory of Optical Detection Technology for Oil and Gas, College of Science, China University of Petroleum, Beijing 102249, China

**Keywords:** hierarchical pores, 3DOMM structure, PtSn, propane non-oxidative dehydrogenation

## Abstract

Herein, the hierarchical porous catalyst of 3-dimensional ordered macro-mesoporous (3DOMM) Al_2_O_3_ supported active PtSn nanoparticles (NPs) was prepared by the combined synthesized path of evaporation-induced self-assembly with colloid crystal template (EISA-CCT) methods. The hierarchical macro-mesoporous composite structure can markedly increase the specific surface area, accommodate the diffusion of propene, and decrease the number of surface acid sites. In addition, the special surface property and pore structure of 3DOMM-Al_2_O_3_ can modify the interaction between metals and substrates, as well as stabilize the metal nanoparticle, which promotes the formation of a highly active and stable PtSn phase. The PtSn/3DOMM-Al_2_O_3_ catalyst exhibits higher productivity and stability than PtSn/Al_2_O_3_ catalysts with macropore and mesopore structures. The PtSn/3DOMM-Al_2_O_3_ catalyst displays the best catalytic performance with propylene selectivity over 95% at a propane conversion of 33.9%. The study of the ordered hierarchical porous structure of PtSn/3DOMM-Al_2_O_3_ catalysts can contribute to obtaining improved catalysts in industrial processes.

## 1. Introduction

Propylene is a significantly important hydrocarbon compound that contributes to the intermediates of a variety of chemical products and is widely used in human production and life [1]. In recent years, thermal and catalytic cracking technologies, which are the traditional production processes of propylene, have no longer been able to meet the increasing needs of the international market [2,3]. Producing propylene through the propane non-oxidative dehydrogenation (PDH) process has become a promising and effective approach. Furthermore, cheap propane is supplied due to the recently increased exploitation of shale gas [4,5]. Although there is keen industrial interest in PDH technology, the dehydrogenation reaction still needs to operate at a high temperature of about 650 °C to break the stable C-H bond of alkane [6,7]. The harsh reaction condition could cause many problems for Pt-based catalysts, such as coke deposition and the sintering of metal nanoparticles [8]. The development of Pt-based catalysts with improved yield, selectivity, and stability is the key issue in improving the industrial utilization value of PDH reactions.

Although the Pt element reveals high activity in breaking the C-H bond, the Pt-based catalysts without any promoters still have a high tendency to side reactions such as hydrogenolysis, cracking, and coke deposition and sintering during the reaction process [9,10,11,12]. Comparing pure Pt-based catalysts, the selectivity and stability of PDH can be significantly improved in PtSn-based catalysts. The Sn element could be reduced by hydrocarbon and alloyed with Pt to form PtSn nanoparticles. Pt is the active species of the PtSn-based catalyst, while Sn acts as the promoter [13,14,15,16]. The structural effects and electronic interaction of the promoter on Pt can effectively inhibit the side reactions [17]. In addition, Sn species can effectively disperse metal nanoparticles and reduce the loading amount of precious metal in Pt-based catalysts. Previous studies have also found that Sn species in an Al_2_O_3_ support can inhibit the sintering of PtSn alloy particles during regeneration [18]. The surface properties of the support are highly related to the active site structure of the PtSn-based catalyst. The dispersion of metal nanoparticles and the PtSn alloying phase in the catalyst is also dependent on the features of the interaction between the metal and the support [19,20]. Conventional supports for PtSn-based catalysts for PDH include Al-based, Si-based, and Zr-based oxide and carbon materials. Both the activity and deactivation rate during the PDH reaction highly depends upon the structure-property of the support.

Al_2_O_3_ with transition phases such as θ phase, η phase, and γ phase is widely applied in many Pt-based catalysts as a kind of supporting material due to its large surface area and high thermodynamic stability [21]. Among them, γ-Al_2_O_3_ is the most important phase of the PtSn-based catalyst for a PDH reaction [22]. γ-Al_2_O_3_ possesses the highest surface area, but the lowest thermodynamic stability compared with other transition Al_2_O_3_, which results in complex surface properties. The transition state of the γ phase provides a high degree of freedom for atomic arrangement, which allows Al^3+^ ions to locate among the tetrahedral, pentahedral, and octahedral sites in Al_2_O_3_, in order that the structures adopt an intangible arrangement and disorder. Therefore, fundamental research about the surface of γ-Al_2_O_3_ and strong support-metal interaction (SMI) are highly debated subjects of abundant ongoing questions related to several areas, including the lattice defect, phase crystallography, and mesoscopic morphology structure [23]. For example, the γ-Al_2_O_3_ nanosheet with exposure of the penta-coordinated Al^3+^ sites can better facilitate the anchor of the isolated dispersed metal atoms than γ-Al_2_O_3_ with ordinary morphology [24]. It is worth noting that the morphology of the support also greatly affects the diffusion process of propane and propylene during the PDH process, which affects the activity and stability at high space velocity and the partial pressure of propane under simulated industrial conditions [25]. Therefore, PtSn-based catalytic material with high performance can reasonably be obtained by carefully adjusting the pore structure of γ-Al_2_O_3_.

Herein, PtSn/γ-Al_2_O_3_ catalysts with a controllable pore structure, including ordered mesopore, ordered macropore, and hierarchically ordered macro-mesopore, are prepared by the combined pathway of evaporation-induced self-assembly with colloid crystal templates (EISA-CCT) methods. The 3-dimensional ordered macropores-mesoporous nanostructure (3DOMM) combined with the structural advantages of macropore and mesopore should increase the surface area of support and diffusion for propylene, which inhibits carbon deposition and the other side reactions of propylene in the channel of the catalyst. In addition, the special morphology of a 3DOMM-Al_2_O_3_ support also affects the surface acidity and Al^3+^ coordination structure, which plays an important role in anchoring sites for highly fine PtSn alloy nanoparticles. Therefore, designing and preparing a multistage pore structure for γ-Al_2_O_3_ is helpful to understand the complex structure–activity relationship of alumina and in designing and preparing high-performance propane non-oxidative dehydrogenation catalysts for industrial applications.

## 2. Experimental Section

### 2.1. Catalyst Preparation

**Scheme 1:**
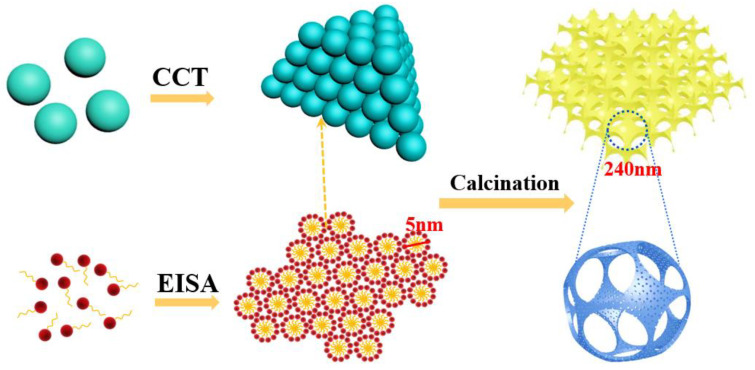
Diagram for the preparation of the 3DOMM-Al_2_O_3_ support.

PtSn/γ-Al_2_O_3_ catalysts with ordered mesopore (M-Al_2_O_3_), 3-dimensional ordered macropore (3DOM-Al_2_O_3_), and 3-dimensional ordered macro-mesopore (3DOMM-Al_2_O_3_) were obtained by varied methods. Polymethyl methacrylate (PMMA) microspheres were tightly packed three-dimensionally (3D) after the centrifugal operation. As shown in Figure S1, the particle size of the microspheres is around 300 nm. These 3D close-packed microspheres were used to obtain the macropore structure. The PMMA microspheres were immersed in an Al precursor solution. The solution fills the interspace between the PMMA microspheres to build structure of ordered macropores. F127 was employed to obtain a mesoporous structure. As the solvent evaporates, F127 molecules tend to form saturated micelles, which arrange themselves into a two-dimensional hexagon (p6mm) ordered pattern. During the growth process, Al ions adhere to the template to build ordered mesopores. After drying and calcining, the template (F127 and PMMA) can be removed and the ordered pores (macropore and mesopore) structure is obtained. As shown in Scheme 1, the 3DOMM nanostructured catalyst was obtained by the EISA-CCT method using two templates, while the 3DOMM structure was obtained after drying and calcining. For all the catalysts, the templates (F127 micelles or PMMA microspheres) were removed in a tube furnace under air (50 mL min^−1^) at 300 °C for 3 h, and then the temperature was increased to 650 °C for 4 h to crystallize the alumina. After obtaining γ-Al_2_O_3_ with different morphologies, Pt species (0.5 wt%) and Sn species (1 wt%) were added into the support by incipient wetness impregnation to obtain the PtSn/γ-Al_2_O_3_ catalyst. A detailed description of the synthesis pathway is listed in part of the Supporting Information.

### 2.2. Characterization

Scanning electron microscope (SEM) graphs were observed using a ZEISS Gemini SEM 300 microscope. Transmission electron microscope (TEM) images were obtained and energy dispersive spectrometer (EDS) element mapping was performed using the LaB6 2100 electron microscope.

The N_2_ adsorption-desorption results were obtained using Micromeritics TriStar-Ⅱ 3020 equipment. The catalyst was pretreated at 200 °C in a constant temperature tank to remove the adsorbed vapor. The X-ray diffraction (XRD) pattern was investigated with a Bruker Advance spectrometer.

The ^27^Al nuclear magnetic resonance (NMR) spectra were obtained using an Agilent 600 DD2 spectrometer. The temperature-programmed desorption of NH_3_ (NH_3_-TPD) was recorded using Autochem-II 2920 equipment. The catalyst (0.1 g) was placed in a quartz reactor. The catalyst was pretreated with He at 300 °C for 1 h and cooled to 50 °C, then treated with 10% NH_3_ balanced with He (30 mL min^−1^) for 1 h and then purged with pure He (30 mL min^−1^) for 1 h. The temperature was raised to 700 °C with a heating rate of 10 °C min^−1^. The NH_3_ desorption signal was observed using a thermal conductivity detector (TCD). The pyridine infrared adsorption (Py-IR) results were given by the Nicolet 6700 Fourier Transform Infrared spectrometer. Before the test, the catalysts were dried for 1 h at 300 °C.

The temperature-programmed reduction of the H_2_ (H_2_-TPR) pattern was conducted on a homemade fixed-bed reactor. The catalyst (0.1 g) was dried at 300 °C for 1 h, and then cooled to 50 °C under N_2_. Then, the H_2_ balanced by N_2_ (10%, 50 mL min^−1^) went through the bed at a temperature of 50–750 °C with a heating rate of 10 °C min^−1^. The consumption signal was recorded by a TCD. The temperature-programmed desorption of H_2_ (H_2_-TPD) was conducted using similar equipment with H_2_-TPR. The catalyst (0.1 g) was first dried for 1 h at 300 °C and reduced in flowing H_2_ (pure, 30 mL min^−1^) at 600 °C for 2 h. Then, the catalyst was cooled to room temperature and adsorbed in H_2_ for 1 h. The temperature was increased to 700 °C with a heating rate of 10 °C min^−1^ and the desorption signal was recorded by the TCD.

The differential thermal analysis and thermal gravimetric analysis (TG-DTA) were conducted in a homemade reaction instrument. The temperature was increased from room temperature to 800 °C at a rate of 10 °C min^−1^ under air (30 mL min^−1^). Raman patterns were obtained with Renishaw inVia reflex Raman equipment with excitation wavelengths of 532 nm.

### 2.3. Evaluation of Catalytic Performance

The PDH tests were conducted in a quartz tubular micro-reactor. The catalyst (0.2 g, 40–60 meshes) was loaded into the reactor. Before the catalytic evaluation, the catalyst was pretreated with H_2_ (pure, 10 mL min^−1^) for 2 h at 500 °C. The PDH reaction was carried out at 600 °C. The propane was fed at a weight hourly space velocity (WHSV) of 3.0 h^−1^ (C_3_H_8_/H_2_ = 1/1). Then, the products were analyzed using online gas chromatography (GC) equipped with a flame ionization detector (FID) and a TCD. The conversion and selectivity were obtained as follows:

$$\mathrm{Conversion}\left(\%\right)=\frac{C3H8\;\mathrm{reacted}}{\;C3H8\;\mathrm{fed}\;}\;\text{×}\;100\%$$

$$\mathrm{Selectivity}\left(\%\right)=\frac{C3H6\;\mathrm{fromed}\;}{C3H8\;\mathrm{reacted}}\;\text{×}\;100\%$$

## 3. Results and Discussion

### 3.1. Physical Properties and Morphology

**Figure 1:**
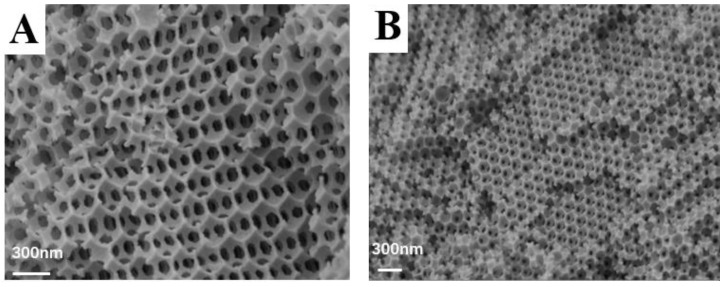
SEM images of (**A**) PtSn/3DOM-Al_2_O_3_ catalyst and (**B**) PtSn/3DOMM-Al_2_O_3_ catalyst.

The morphology of the PMMA microspheres and macropore catalysts was identified by SEM. As shown in Figure S1, the PMMA microspheres are closely packed and form a uniform interspace between them. Tightly arranged and uniform PMMA microspheres are important for the formation of ordered macropores. During the EISA-CCT process, Al ions hydrolyze, condense, and crystallize in the interspace of PMMA microsphere templates to form a macropore structure. As shown in Figure 1A,B, the PtSn/3DOM-Al_2_O_3_ and PtSn/3DOMM-Al_2_O_3_ catalysts exhibit well-defined and uniform 3D-ordered macropore structures. The average diameter of the macropore (about 240 nm) is shrunken compared with that of the PMMA microsphere templates (about 300 nm), which can be attributed to the melting of the PMMA microspheres and the crystallization of the Al_2_O_3_ during the calcination process [26]. Notably, some areas in the macropore also display disorder and a broken structure, which can be attributed to the mechanical milling which will inevitably damage part of the porous structure during the preparation of the SEM sample.

**Figure 2:**
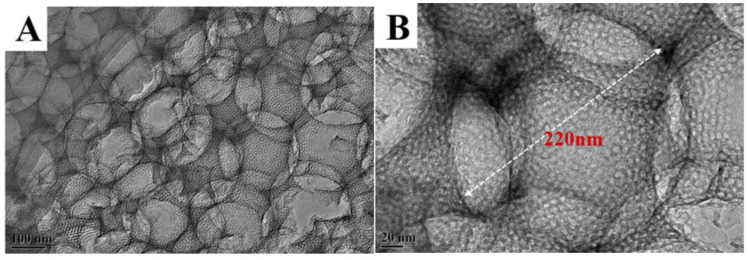

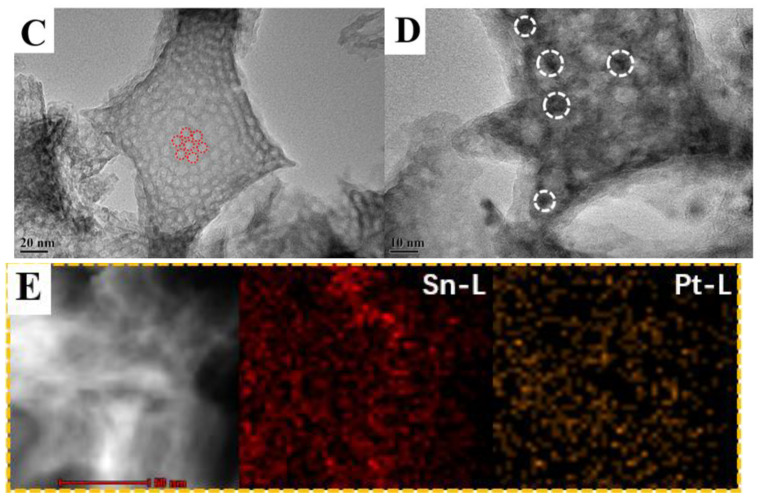
TEM images of fresh (**A**–**C**) and reduced (**D**) PtSn/3DOMM-Al_2_O_3_ catalyst; (**E**) EDS element-mapping analyses of PtSn/3DOMM-Al_2_O_3_ catalyst.

The mesoporous structures and supported metal nanoparticles (NPs) were identified with a TEM. As shown in Figure 2A,B, the PtSn/3DOMM-Al_2_O_3_ sample shows an ordered macropore structure. The ordered macropores are approximately 220 nm in pore size, and the thickness of the Al_2_O_3_ wall is approximately 80 nm, which matches the SEM image. In addition, there are many ordered mesopores present on the macropore wall of the PtSn/3DOMM-Al_2_O_3_ catalyst (Figure 2C). Ordered mesopores with a diameter of about 8–9 nm are densely arranged in a hexagon region on the macropore wall of 3DOMM Al_2_O_3_, indicating the hierarchical macro-mesoporous composited structure is successfully synthesized by the EISA-CCT method. The highly dispersed metal nanoparticles are formed on the surface of 3DOMM-Al_2_O_3_ with a uniform particle size of around 3 nm (Figure 2D). The atomic number of the Pt element is much larger than the Al element. Therefore, the metal NPs can be attributed to the Pt or PtSn alloy, while the nearly colorless parts can be attributed to the mesopores. For identifying the distribution of metal on the support, an EDS was employed. The elemental mapping images show Pt and Sn elements evenly distributed on the surface of the catalysts, indicating the formation of a PtSn alloy. In addition, TEM images of the PtSn/3DOM-Al_2_O_3_ and PtSn/M-Al_2_O_3_ catalysts are shown in Figure S2. The PtSn/M-Al_2_O_3_ catalyst has a mesopore structure with an average size of 10 nm, while the PtSn/3DOM-Al_2_O_3_ catalyst only has a macropore structure.

**Figure 3:**
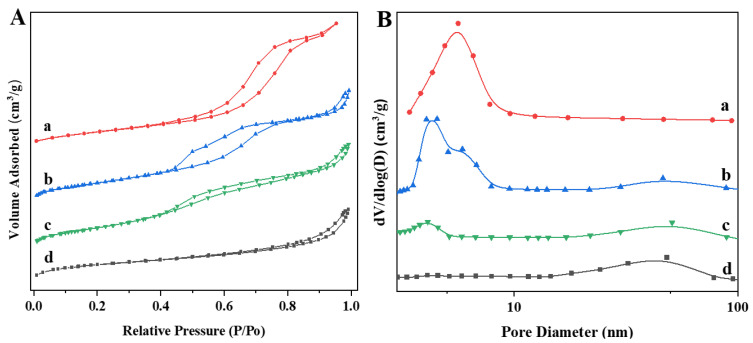
(**A**) N_2_ adsorption–desorption isotherms, (**B**) pore size distribution curves. (a) M-Al_2_O_3_; (b) 3DOMM-Al_2_O_3_; (c) PtSn/3DOMM-Al_2_O_3_; (d) 3DOM-Al_2_O_3_.

N_2_ adsorption–desorption experiments were applied to obtain the textual structure of catalysts. As shown in Figure 3A, the M-Al_2_O_3_ support shows an H1 hysteresis loop with the IV-type isotherm, which is characteristic of an ordered mesoporous nanostructure. The M-Al_2_O_3_ support has a specific surface area of 347 m^2^ g^−1^. The 3DOM-Al_2_O_3_ support shows the H3 hysteresis loop attributed to the ordered macropore structure. The specific surface area of the PtSn/3DOM-Al_2_O_3_ sample is 273 m^2^ g^−1^. Due to the presence of a hierarchical macro-mesoporous composite structure, the 3DOMM-Al_2_O_3_ material possesses the highest specific surface area of 369 m^2^ g^−1^ [27]. After the impregnation process, the specific surface area of the PtSn/3DOMM-Al_2_O_3_ sample is still maintained at 346 m^2^ g^−1^, indicating that the impregnated metal is evenly dispersed on the support. As shown in Figure 3B, the M-Al_2_O_3_ and 3DOMM-Al_2_O_3_ supports represent an even pore size distribution from 5 to 10 nm, which can be assigned to the mesopore structure. Furthermore, there is an additional pore distribution above 50 nm for the 3DOM-Al_2_O_3_ and 3DOMM-Al_2_O_3_ supports, which can be attributed to the macropore structure. The impregnation of PtSn NPs causes an obvious decrease in the pore distribution of mesopores, confirming that PtSn NPs are mainly dispersed in mesopores.

**Figure 4:**
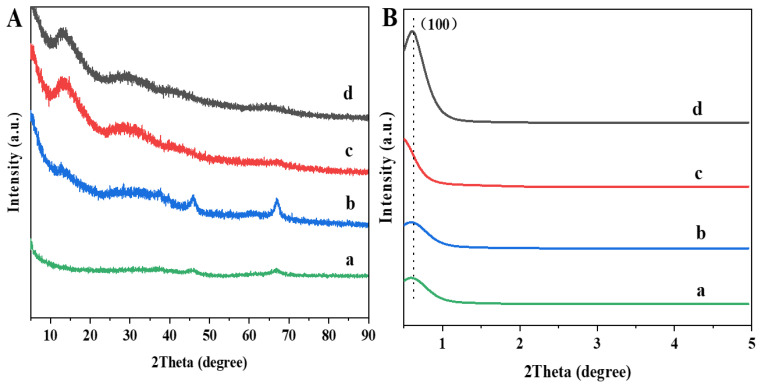
Wide- and low-angle XRD patterns (**A**,**B**). (a) 3DOMM-Al_2_O_3_; (b) PtSn/3DOMM-Al_2_O_3_; (c) PtSn/3DOM-Al_2_O_3_; (d) PtSn/M-Al_2_O_3._

Wide-angle XRD measurements were employed to investigate the crystal phase of the PtSn/Al_2_O_3_ catalysts. As shown in Figure 4A, the PtSn/M-Al_2_O_3_, PtSn/3DOM-Al_2_O_3_, and PtSn/3DOMM-Al_2_O_3_ catalysts exhibit characteristic diffraction peaks at 31.9°, 39.4°, 45.8°, and 66.8°, which are attributed to (220), (222), (400), and (440) facets (PDF#79-1558) of Al_2_O_3_, respectively. No characteristic peaks corresponding to Pt and Sn were observed, which is due to the highly dispersed Pt-Sn NPs. In addition, low-angle XRD was employed to verify the ordered mesoporous nanostructure. As shown in Figure 4B, PtSn/M-Al_2_O_3_ and PtSn/3DOMM-Al_2_O_3_ catalysts exhibit a diffraction peak of 2θ at 0.6°, which is attributed to the (100) facet of the 2D hexagonal (p6mm) of the ordered mesopore nanostructure [28]. For the PtSn/3DOM-Al_2_O_3_ catalysts, the characterized diffraction peak is not located at the same position, indicating an irregular pore structure. In sum, the 3DOMM-Al_2_O_3_ support has a hierarchical macro-mesoporous composite structure, while the PtSn NPs highly disperse in the mesopores of the PtSn/3DOMM-Al_2_O_3_ catalyst.

### 3.2. Surface Property of Support

**Figure 5:**
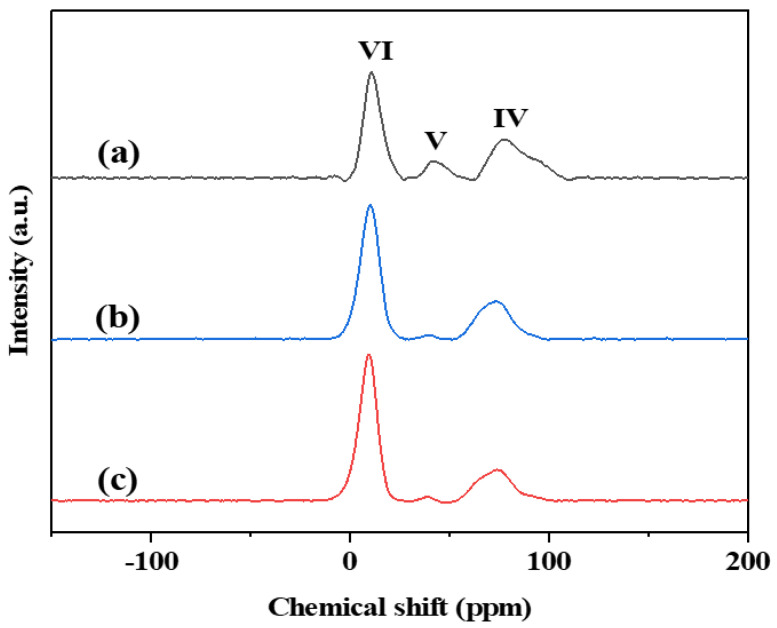
NMR patterns of the catalysts:(a) PtSn/3DOMM-Al_2_O_3_; (b) PtSn/3DOM-Al_2_O_3_; (c) PtSn/M-Al_2_O_3_.

To perform an in-depth investigation of the surface characteristics of the Al_2_O_3_ support, solid-state ^27^Al NMR techniques were employed. Figure 5 exhibits the NMR spectra of the PtSn/Al_2_O_3_ catalysts. Three resonance signals were observed at 73, 44, and 9 ppm, which were assigned to the Al^3+^ ion in the tetra-coordinated (AlO_4_), penta-coordinated (AlO_5_), and hexa-coordinated (AlO_6_) coordination, respectively [29]. Penta-coordinated AlO_5_ is universally acknowledged to be the site for anchoring metal sites due to its coordination of unsaturated properties. For Pt/γ-Al_2_O_3_ catalysts, coordination unsaturated penta-coordination (AlO_5_) is the main site for stabilizing Pt [30]. The PtSn/3DOMM-Al_2_O_3_ catalyst has a much greater proportion of penta-coordinated Al^3+^ ions on its surface than both the PtSn/M-Al_2_O_3_ and PtSn/3DOM-Al_2_O_3_ catalysts. In addition, the proportions of Al^3+^ ions with different coordination sites are exhibited in Table 1. The PtSn/3DOMM-Al_2_O_3_ catalyst has the highest fraction of penta-coordinated Al^3+^ ions at 9%, while the PtSn/M-Al_2_O_3_ and PtSn/3DOM-Al_2_O_3_ catalysts have only about 1%. The penta-coordinated Al^3+^ ion sites on the PtSn/3DOMM-Al_2_O_3_ catalyst can enhance the anchoring interaction between γ-Al_2_O_3_ and Pt, which avoids the aggregation of nanoparticles and improves the stability.

**Figure 6:**
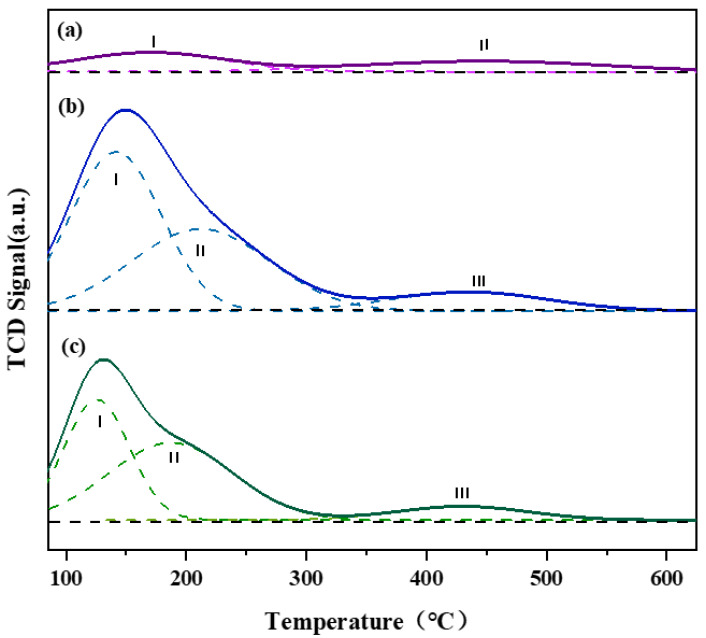
NH_3_-TPD patterns of the PtSn/γ-Al_2_O_3_: (a) PtSn/3DOM-Al_2_O_3_; (b) PtSn/M-Al_2_O_3_; (c) PtSn/3DOMM-Al_2_O_3_.

The acid property of the varied catalysts was studied using NH_3_-TPD experiments, and the corresponding NH_3_-TPD patterns are shown in Figure 6. It is clear that two ammonia desorption peaks can be observed in the PtSn/3DOM-Al_2_O_3_ catalysts. The NH_3_-TPD profiles of the PtSn/3DOMM-Al_2_O_3_ and PtSn/M-Al_2_O_3_ catalysts display three desorption peaks at about 130, 200, and 430 °C. The PtSn/M-Al_2_O_3_ catalyst reveals the highest amount of acid sites. To obtain the relative amount of acid sites and the acidic strength, a Gaussian peak fitting was conducted to obtain the deconvolution of the NH_3_-TPD results. The fitting results are shown in Figure 6 and Table 2. It is widely known that the NH_3_ desorption in the temperature regions of 120–200, 200–350, and 350–450 °C can be attributed to the weak acid sites, medium acid sites, and strong acid sites, respectively [31]. The area percentages of the three different peaks of the PtSn/3DOMM-Al_2_O_3_ catalyst are 41, 50, and 9%, respectively. For the PtSn/M-Al_2_O_3_ sample, the area percentages are 51, 39, and 10%, respectively. The PtSn/3DOM-Al_2_O_3_ catalyst only shows two peaks at 169 and 447 °C, and their area percentages are 53 and 47%, respectively. Based on the result of Al NMR and NH_3_-TPD, the pore structure and morphology of the Al_2_O_3_ support plays a primary role in the surface property. In addition, the side reactions of the PDH can be catalyzed by Lewis acid sites in Al_2_O_3_. Thus, it could be inferred that side reactions occur more easily on the support surface of the PtSn/M-Al_2_O_3_ catalyst with a high amount of acid sites than that of the PtSn/3DOMM-Al_2_O_3_ catalyst [32].

**Figure 7:**
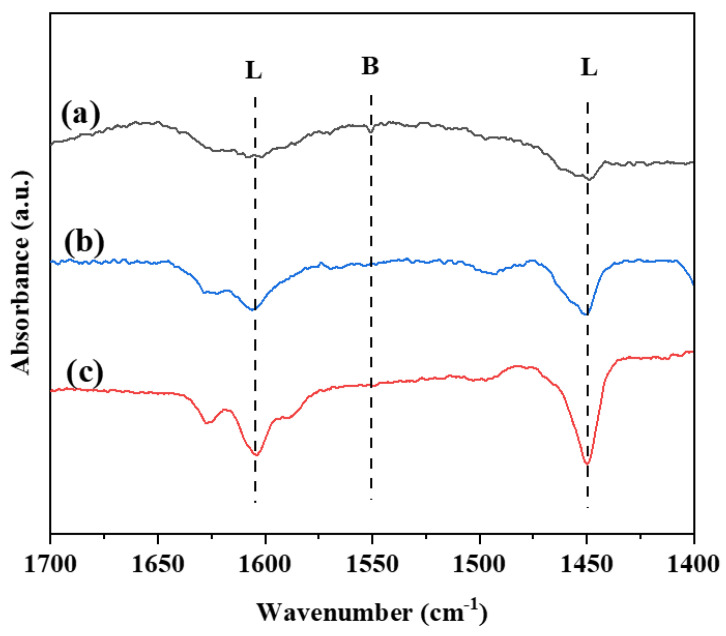
Py-IR patterns of the PtSn/Al_2_O_3_ catalysts: (a) PtSn/3DOM-Al_2_O_3_; (b) PtSn/3DOMM-Al_2_O_3_; (c) PtSn/M-Al_2_O_3_.

Figure 7 exhibits the IR pattern of the three samples after the adsorption of pyridine (Py). The PtSn/3DOM-Al_2_O_3_ catalyst shows a very weak IR peak located at 1550 cm^−1^, which can be attributed to the peak of the Brønsted acid site. Moreover, there is no Brønsted acid site in the PtSn/M-Al_2_O_3_ and PtSn/3DOMM-Al_2_O_3_ catalysts [33]. The Py molecules can be also adsorbed by the Lewis acid site. The IR peak at 1595 cm^−1^ is due to the Py molecule adsorbed at the weak Lewis acidic site. The IR peak at 1605 cm^−1^ can be attributed to the Py molecule adsorbed at the Lewis acid site with medium strength. In addition, the IR peak located at 1626 cm^−1^ can be attributed to strong Lewis acid sites [34]. PtSn/M-Al_2_O_3_ catalysts show an obviously higher amount of Lewis acid sites than PtSn/3DOMM-Al_2_O_3_ catalysts, which can be attributed to the pore-dependent surface property of Al_2_O_3_. In summary, the catalysts with M-Al_2_O_3_, 3DOM-Al_2_O_3_, and 3DOMM-Al_2_O_3_ support reveal different surface properties. The PtSn/3DOMM-Al_2_O_3_ catalyst possesses a lower amount of acid sites compared to the PtSn/M-Al_2_O_3_ catalyst, which can inhibit side reactions such as coke deposition and other side reactions during the PDH reaction. In addition, the varied amount of penta-coordinated AlO_5_ sites in γ-Al_2_O_3_ also infers a different interaction between support with metal, which can affect the dispersion and structure of supported PtSn active species.

### 3.3. Structure of Active Sites

**Figure 8:**
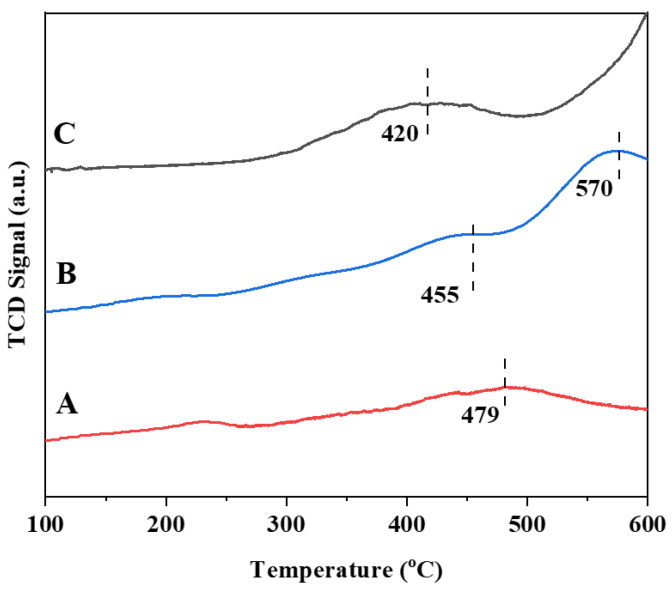
H_2_-TPR pattern of the PtSn/Al_2_O_3_ catalysts. (A) PtSn/M-Al_2_O_3_; (B) PtSn/3DOMM-Al_2_O_3_; (C) PtSn/3DOM-Al_2_O_3_.

The nanopore structure and surface chemical property of catalysts can closely affect the Pt-Sn interaction and dispersion of metal nanoparticles. Therefore, H_2_-TPR measurements were employed to test the redox property of the PtSn/γ-Al_2_O_3_ samples, which is shown in Figure 8. The peak at about 200 °C is the reduction peak of the Pt component. During the reduction, the metallic state (Sn^0^) can be reduced to form the PtSn alloy, in which Sn can increase the reduction temperature of Pt. All the PtSn/γ-Al_2_O_3_ catalysts show a reduction peak located at around 400 °C, which is attributed to the Pt-Sn alloy formation [35,36]. Notably, the reducibility of catalysts is highly dependent on the surface chemical property of the support. The reduction temperatures of the PtSn/M-Al_2_O_3_ and PtSn/3DOMM-Al_2_O_3_ catalysts are located at 479 and 455 °C, which are higher than the reduction temperature of the PtSn/3DOM-Al_2_O_3_ catalyst (420 °C). This increased reduction temperature is attributed to a stronger interaction between the metal and the support, which infers a higher dispersion of metal species. In addition, the PtSn/3DOMM-Al_2_O_3_ and PtSn/3DOM-Al_2_O_3_ catalysts show a reduction peak at a temperature above 500 °C, which is attributed to the biggish SnO_x_ nanoparticle weakly anchored by γ-Al_2_O_3_, indicating that the macropore nanostructure would affect the dispersion of metal. This can be attributed to the varied geometric confining effect of the macropores and mesopores on the loaded species. The macropore structure will lead to an increase in metal particle size and a decreased interaction between the metal and the carrier. For 3DOMM materials, the abundant mesoporous are conducive to the formation of small PtSn NPs through the confining effect of mesopores.

**Figure 9:**
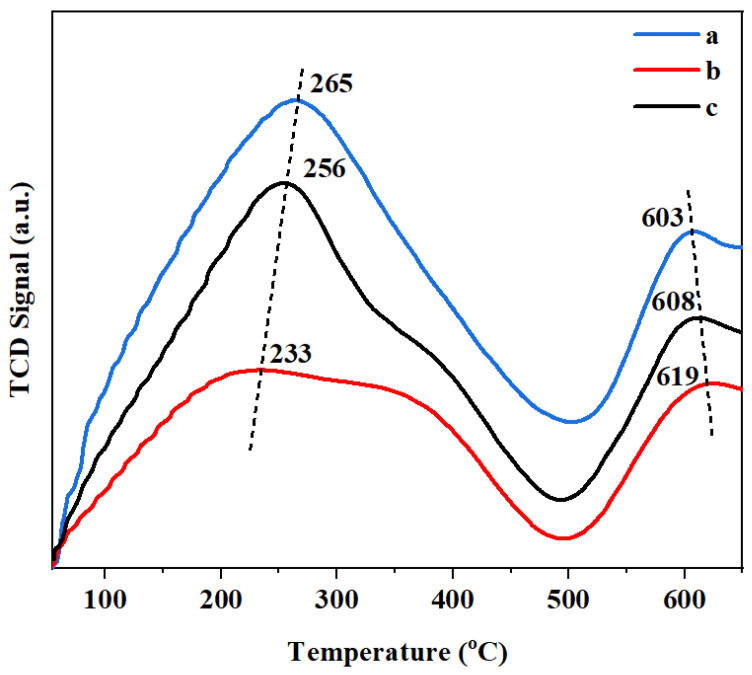
H_2_-TPD patterns of the PtSn/Al_2_O_3_ catalysts: (a) PtSn/3DOMM-Al_2_O_3_; (b) PtSn/M-Al_2_O_3_; (c) PtSn/3DOM-Al_2_O_3_.

In order to analyze the dispersion of the PtSn species more thoroughly, the H_2_-TPD measurement is further employed. The H_2_-TPD patterns of the different samples are shown in Figure 9, while two desorption peaks can be detected in the patterns. It is well known that H_2_ could dissociate into two H atoms on the surface of metal active sites. Based on this, the low-temperature desorption peak (below 500 °C) is assigned to chemisorbed H atoms removed from the metal nanoparticles [37,38,39]. For the PtSn/3DOMM-Al_2_O_3_ catalyst, it displays the highest amounts of adsorbed hydrogen, which indicates that PtSn nanoparticles are highly dispersed on 3DOMM-Al_2_O_3_ after reduction. The PtSn/3DOMM-Al_2_O_3_ demonstrates a much larger peak area of H adsorption than the PtSn/3DOM-Al_2_O_3_ sample, indicating that the Pt active sites are highly dispersed on the 3DOMM-Al_2_O_3_ support. In addition, after chemical adsorption, the H atoms on the surface of Pt can also overflow the adjacent support, which is also known as hydrogen spillover. The high-temperature desorption peak above 600 °C can be attributed to the desorption peak of spilled H atoms [40]. Overall, the pore structure of γ-Al_2_O_3_ strongly affects the dispersion of the PtSn active sites. Strong metal-support interactions (SMSI) and the confined effect of mesopores in the macropore walls can result in small PtSn nanoparticles forming.

### 3.4. Catalytic Performance for PDH

**Figure 10:**
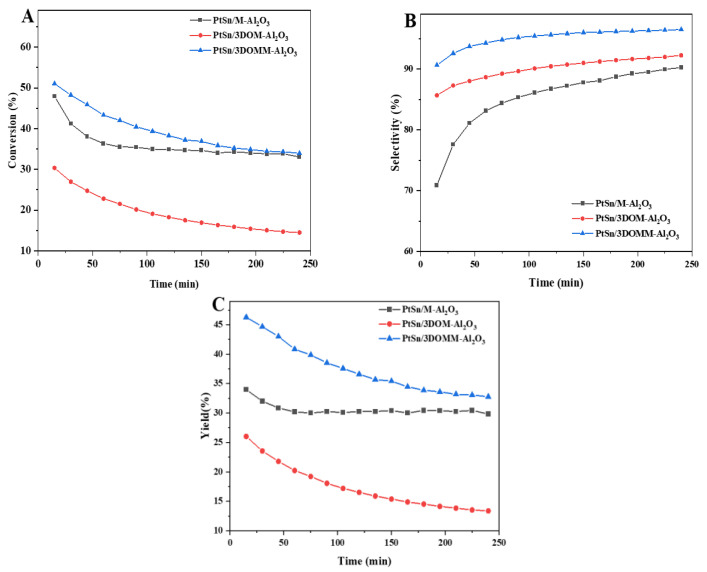
Catalytic performance for a propane dehydrogenation reaction. (**A**) conversion, (**B**) selectivity, and (**C**) yield.

The catalytic performances of the PtSn/γ-Al_2_O_3_ catalyst are shown in Figure 10A,B, which shows changes in conversion and selectivity with operating time, respectively. Among all the PtSn/Al_2_O_3_ catalysts, the PtSn/3DOMM-Al_2_O_3_ catalyst shows the most desired catalytic performance. The initial conversion rate of 51.0% reduced to a conversion rate of 33.9% after 4 h of reaction, and the selectivity increased from 90.7% to 96.5%. The PtSn/M-Al_2_O_3_ catalyst reveals a high initial conversion rate of propane (47.9%) but a low initial selectivity of propylene (initial: 70.9%), and it quickly deactivates within the first hour. However, the conversion rate dropped to 33.0% after 4 h, while the selectivity of propylene gradually increased (final: 90.3%). Although the conversion rate of PtSn/3DOM-Al_2_O_3_ is the lowest (initial: 30.4%, final: 14.5%), its selectivity (initial: 85.7%, final 92.3%) is higher than that of PtSn/M-Al_2_O_3_. This phenomenon can be explained by the volcanic curve relationship between selectivity and conversion in a PDH reaction [41]. Compared with PtSn/γ-Al_2_O_3_ catalysts with other porous structures, the hierarchical macro-mesoporous PtSn/3DOMM-Al_2_O_3_ catalyst has a higher conversion, selectivity, and strong stability. As shown in Figure 10C, it is also obvious that the PtSn/3DOMM-Al_2_O_3_ catalyst possesses the highest propylene yield, followed by PtSn/M-Al_2_O_3_, and the PtSn/3DOM-Al_2_O_3_ catalysts have the lowest yield during a 4 h reaction test. The PtSn/3DOMM-Al_2_O_3_ catalyst displays better comprehensive conversion and selectivity than the PtSn/M-Al_2_O_3_ and PtSn/3DOM-Al_2_O_3_ catalysts. In addition, the catalytic performance of the PtSn/3DOMM-Al_2_O_3_ catalyst was compared with that of Pt-based catalysts reported recently. As shown in Table S1, the PtSn/3DOMM-Al_2_O_3_ catalyst has high conversion and selectivity, indicating that it has a good prospect for industrial PDH application.

In order to improve the gas–solid contact area, catalysts for alkane dehydrogenation reactions are often designed with mesoporous structures. However, a small mesoporous pore size will cause the slow desorption of propylene, coke deposition, and blocked pores. For the 3DOMM-Al_2_O_3_ support, the PtSn active sites are mainly located in the mesoporous pores of the macropore walls, which effectively improves the pore connectivity and pore volume, facilitates the diffusion of propylene from pores and improves the carbon-holding capacity of the PtSn/γ-Al_2_O_3_ catalyst [42]. Therefore, the PtSn/3DOMM-Al_2_O_3_ catalyst shows the high stability for the PDH reaction. The specific surface area of the 3DOMM-Al_2_O_3_ material is also significantly higher than the specific surface area of the 3DOM-Al_2_O_3_ material due to the abundant mesopores in the macropore walls, which is beneficial to the dispersion of PtSn-active species. In addition, the hierarchical macro-mesoporous structure also affects the surface chemical property of γ-Al_2_O_3_. The catalytic dehydrogenation reaction performance of the catalyst is closely related to the chemical property of active metal, while acid sites on γ-Al_2_O_3_ are important to side reactions [43]. For the PtSn/M-Al_2_O_3_ catalyst, the amount of acid sites is relatively higher than the PtSn/3DOM-Al_2_O_3_ and PtSn/3DOMM-Al_2_O_3_ catalysts, consequently reducing the reaction selectivity and stability. In addition, for the PtSn/3DOMM-Al_2_O_3_ catalyst, the PtSn nanoparticles have a higher dispersion than that of the PtSn/3DOM-Al_2_O_3_ catalyst. Through the characterization of H_2_-TPR and H_2_-TPD, it can be concluded the interaction between the 3DOMM-Al_2_O_3_ support and PtSn component is beneficial to the formation of ultrafine Pt-Sn alloy particles. The propane dehydrogenation reaction is structurally insensitive, and its side reactions are structurally sensitive. The smaller metal nanoparticles can inhibit side reactions, which also increase the stability of the PtSn/3DOMM-Al_2_O_3_ catalyst [44]. Therefore, the PtSn/3DOMM-Al_2_O_3_ catalyst shows the best catalytic performance among all the PtSn/γ-Al_2_O_3_ catalysts.

**Figure 11:**
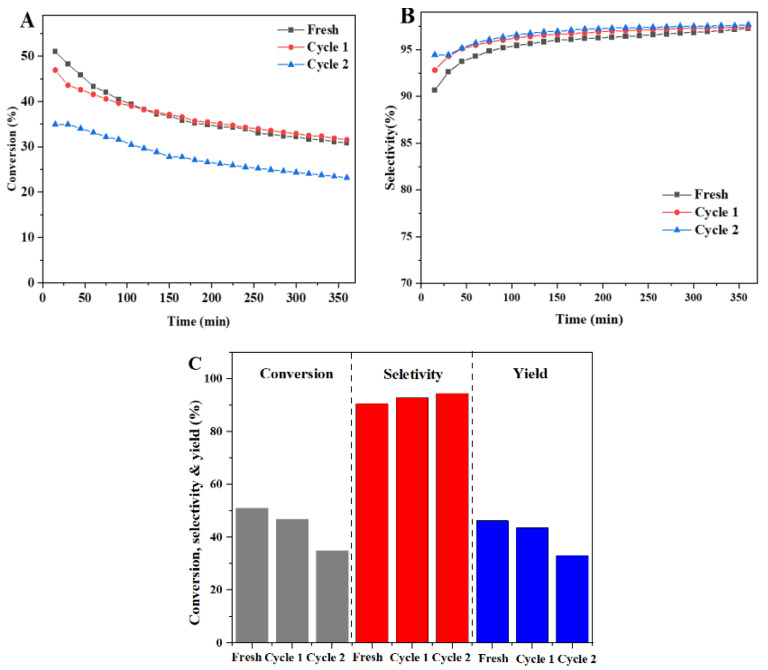
(**A**) Conversion and (**B**) selectivity of the regenerated PtSn/3DOMM-Al_2_O_3_ catalyst. Conversion, selectivity, and yield change with the number of regenerations (**C**).

To evaluate the regeneration performance of the PtSn/3DOMM-Al_2_O_3_ catalyst, a reaction activity test was carried out using a circulating reaction catalyst. As shown in Figure 11A, the initial propane conversion rate slightly decreased after the catalyst was first regenerated. However, in the second cycle reaction, the propane conversion rate was reduced by about 16% compared with the fresh catalyst. This may be due to the sintering of Pt nanoparticles after treatment with air at high temperatures; the increased selectivity is caused by the reduction of propene in reaction gas due to the decreased conversion (Figure 11B) [45]. In Figure 11C the detailed changes in conversion, selectivity, and yield are listed in a bar graph. These results indicate that the propylene selectivity of the PDH reaction remained stable for the PtSn/3DOMM-Al_2_O_3_ catalyst after regeneration. In summary, the 3DOMM-Al_2_O_3_ as a good support improves both the physical and chemical properties of PtSn/γ-Al_2_O_3_ catalysts, which can improve the catalytic performance of the PDH reaction.

### 3.5. Coke Analysis of Used Catalysts

**Figure 12:**
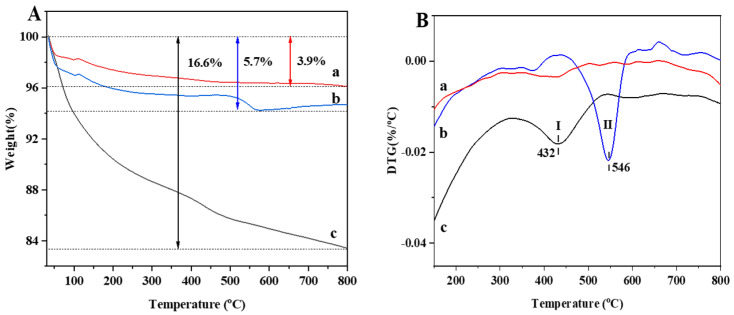
TG (**A**) and DTG (**B**) patterns of the PtSn/Al_2_O_3_ catalysts: (a) PtSn/3DOM-Al_2_O_3_; (b) PtSn/3DOMM-Al_2_O_3_; (c) PtSn/M-Al_2_O_3_.

The amount of coke was quantitatively obtained by TG-DTG, and the mass loss patterns are exhibited in Figure 12A. The total amount of coke follows the order PtSn/3DOM-Al_2_O_3_ (3.9%) < PtSn/3DOMM-Al_2_O_3_ (5.7%) < PtSn/M-Al_2_O_3_ (16.6%). Generally speaking, in the DTG curve, two types of combustion peaks of coke will appear on the Pt-based catalyst. The lower temperature (around 460 °C) peak is assigned to the combustion peak of coke deposition on the around metal nanoparticles. The higher temperature (around 560 °C) peak is attributed to the carbon deposits on the surface of the support [46,47]. It can be seen from Figure 12B that the peak I and peak II appearing in the DTG curve correspond to the carbon deposition on the around metal (peak I) and the coke on the support (peak II), respectively. The peak I at 432 °C for PtSn/M-Al_2_O_3_ can be considered as the coke deposited on the around Pt nanoparticle, and the peak II located at 546 °C for PtSn/3DOMM-Al_2_O_3_ can be considered as the coke deposited on the Al_2_O_3_ support, which can be attributed to macro-mesopore composite structure can facilitates the migration of carbon from the metal active site inside mesopore to the macropore in carrier. The PtSn/M-Al_2_O_3_ catalyst has the largest amount of carbon deposits, which is consistent with the characterization of acidity. Because of it having more Lewis acidic sites on the M-Al_2_O_3_ support, PtSn/M-Al_2_O_3_ triggers more side reactions than PtSn/3DOMM-Al_2_O_3_. PtSn/3DOMM-Al_2_O_3_ has moderate carbon deposits due to its suitable amount of acid sites. Notably, although PtSn/3DOM-Al_2_O_3_ has a small number of carbon deposits, its catalytic performance for the PDH reaction is poor, thus the low amount of coke can be attributed to its low activity. In sum, the low carbon deposition of the PtSn/3DOMM-Al_2_O_3_ catalyst can be attributed to the large surface area, low amount of acid sites, and highly dispersed Pt active components, which inhibits the carbon deposition. At the same time, the pore structure of the PtSn/3DOMM-Al_2_O_3_ catalyst improves the carbon-holding capacity of the catalyst, which improves catalytic stability.

**Figure 13:**
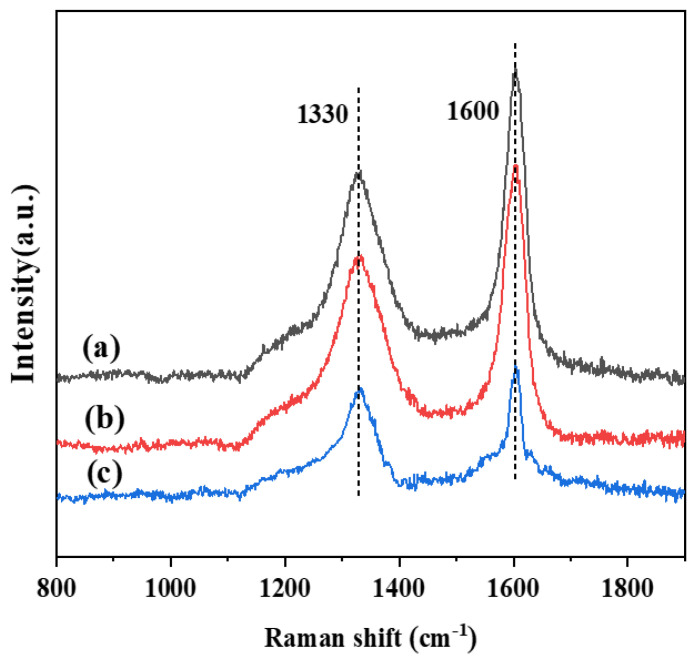
Raman patterns (532 nm) of the PtSn/Al_2_O_3_ catalysts: (a) PtSn/3DOMM-Al_2_O_3_; (b) PtSn/M-Al_2_O_3_; (c) PtSn/3DOM-Al_2_O_3_.

Figure 13 shows the Raman spectra of the used PtSn/Al_2_O_3_ catalysts. The Raman spectrum exhibits two peaks representing the degree of graphitization of the coke, namely 1330 and 1600 cm^−1^. The Raman absorption peaks located at 1330 cm^−1^ and 1600 cm^−1^ are assigned to the D mode and G mode, respectively, which are caused by the multiple aromatic rings of graphite-like carbon materials. Among them, the D mode represents the disordered part of graphitized coking, while the G mode is considered to represent highly graphitized carbon. The ratio of intensity between the D and G modes is generally considered to be an important indicator for evaluating the graphitization degree of carbon material. Generally, a higher intensity ratio between the D and G modes means a lower graphitization degree [48]. The ratio of the intensity of the D to the G peak (I_D_/I_G_) was also obtained and is shown in Table 3. The ratio of I_D_/I_G_ for the PtSn/3DOMM-Al_2_O_3_ catalyst is 0.73, which is much higher than the ratios of I_D_/I_G_ for the PtSn/M-Al_2_O_3_ catalyst (0.66), indicating that the amount of disordered graphitized coke deposited on the PtSn/3DOMM-Al_2_O_3_ catalyst is higher than that on the PtSn/M-Al_2_O_3_ catalyst.

It is widely known that the formation of coke during the PDH reaction involves three processes, while alkene is the main precursor of coke formation: (1) the deep dehydrogenation or cyclization of alkene; (2) the oligomerization of hydrocarbon; (3) a Diels–Alder type reaction [49]. The PtSn/3DOMM-Al_2_O_3_ catalyst has a lower amount of acid sites than the PtSn/M-Al_2_O_3_ catalyst, which inhibits deep dehydrogenation and cyclization by Lewis acid sites. Therefore, the PtSn/3DOMM-Al_2_O_3_ catalyst has a higher I_D_/I_G_ value than the PtSn/M-Al_2_O_3_ catalyst. Notably, the PtSn/3DOM-Al_2_O_3_ catalyst has a much higher I_D_/I_G_ value than the PtSn/3DOMM-Al_2_O_3_ catalyst. This may be due to the low partial pressure of propylene caused by low conversion of PtSn/3DOM-Al_2_O_3_ catalyst which inhibits the deep dehydrogenation process. For the PtSn/M-Al_2_O_3_ catalyst, a high amount of acid sites on the Al_2_O_3_ support resulted in the formation of more graphitized coke deposition. This also proves that the surface acidity of catalysts is an important factor affecting the anti-coke stability of PtSn/γ-Al_2_O_3_ catalysts, which is also consistent with the conclusions of previous studies [50].

## 4. Conclusions

In this work, the effects of supports with different pore structures (3DOM-Al_2_O_3_, M-Al_2_O_3_, and 3DOMM-Al_2_O_3_) on the PDH reaction performance of bimetallic PtSn-based catalysts were studied. The PtSn/3DOMM-Al_2_O_3_ catalyst with a hierarchical porous structure shows the best catalytic performance among these three PtSn/Al_2_O_3_ catalysts. the experimental characterization reveals that the macro-mesoporous composite structure of the PtSn/3DOMM-Al_2_O_3_ catalyst has a significant impact on its physical structure and surface properties. The results indicate that the large specific surface area and hierarchical pore structure of the PtSn/3DOMM-Al_2_O_3_ catalyst are beneficial to the dispersion of PtSn nanoparticles and gas diffusion from the inside of the mesopores. In addition, the difference in the pore structure of supports significantly changes the surface acidity and interaction between the metal and the support. In summary, the design strategy for the hierarchical porous γ-Al_2_O_3_ support is an ideal method for improving the catalytic performance of PtSn-based catalysts in a PDH reaction.

## Figures and Tables

**Table 1:** Fitted results of the NMR experiments of the PtSn/Al_2_O_3_ catalysts.

Catalyst	Peak Area (%)		
	VI	V	IV
PtSn/3DOMM-Al_2_O_3_	52	9	39
PtSn/3DOM-Al_2_O_3_	67	1	32
PtSn/M-Al_2_O_3_	71	1	28

**Table 2:** NH_3_-TPD-fitted results of the PtSn/Al_2_O_3_ catalysts.

Catalyst	T_M_ (°C)			Peak Area (%)		
	I	II	III	I	II	III
PtSn/3DOM-Al_2_O_3_	169	447	/	53	47	/
PtSn/3DOMM-Al_2_O_3_	125	192	430	41	50	9
PtSn/M-Al_2_O_3_	141	211	435	51	39	10

**Table 3:** The I_D_/I_G_ of coked PtSn/Al_2_O_3_ catalysts.

Catalyst	I_D_/I_G_
PtSn/3DOM-Al_2_O_3_	0.84
PtSn/3DOMM-Al_2_O_3_	0.73
PtSn/M-Al_2_O_3_	0.66

## Data Availability

Not applicable.

## Supplementary Materials

The following supporting information can be downloaded at: https://www.mdpi.com/article/10.3390/nano13040728/s1, Figure S1: The morphology of 3D close-packed PMMA templates.; Figure S2: TEM images of 3DOM-Al_2_O_3_ (**A**) and M-Al_2_O_3_ (**B**).; Table S1: Performance comparison of recent Pt-based catalysts. References [18,40,51,52,53] are cited in the Supplementary Materials.
