# Supplementary material for: Ordered Hierarchical Porous Structure of PtSn/3DOMM-Al2O3 Catalyst for Promoting Propane Non-Oxidative Dehydrogenation
Source: Nanomaterials (Basel). 2023 Feb 14;13(4):728. doi: 10.3390/nano13040728 (PMC9959180; doi:10.3390/nano13040728)
Supplement: Supplementary file 1 [file nanomaterials-13-00728-s001.zip › nanomaterials-2196103-supplementary.pdf]

nanomaterials

Supporting Information

Ordered Hierarchical Porous Structure of PtSn/3DOMM-Al_2O_3 Catalyst for Promoting Propane Non-Oxidative Dehydrogenation

Yuanqing Sun [1,†], Bohan Feng [1,†], Qian Lian [1,†], Chengshu Xie [1], Jing Xiong [1,2,*], Weiyu Song [1], Jian Liu [1] and Yuechang Wei [1,2,*]

1 State Key Laboratory of Heavy Oil Processing, College of Science, China University of Petroleum, Beijing 102249, China;
2 Key Laboratory of Optical Detection Technology for Oil and Gas, College of Science, China University of Petroleum, Beijing 102249, China;
* Correspondence: xiongjing@cup.edu.cn (J.X.); weiyc@cup.edu.cn (Y.W.)
† These authors contributed equally to this work.

1. Catalyst Preparation

1.1. Preparation of PMMA template

Monodispersed polymethyl methacrylate (PMMA) microspheres were synthesized through the emulsifier-free emulsion polymerization method. The monomer methyl methacrylate (MMA) (120 mL) and deionized water (240 mL) were added into a four-necked and round-bottomed flask (2000 mL). The MMA solution was heated to 80 ^{o}C in a water bath under Ar gas. Then, 0.3 g potassium persulfate (KPS) was added to the water (40 ml). The obtained KPS solution was heated at 80 ^{o}C for 30 min in a water bath. Then, the KPS solution was added to the MMA solution. The mixture was maintained for 90 min at 80 ^{o}C. Then, the reaction flask was naturally cooled to room temperature and PMMA microspheres were obtained. The solution was filtered and centrifuged at 3000 r min^{-1} for 10 h. The colloidal crystal templates (CCT) were obtained, which were subsequently dried at 50 ^{o}C for 12 h in an oven.

1.2. Preparation of 3DOMM Al_2O_3 support

The typical synthesized pathway for 3DOMM-Al_2O_3 support is listed as follows: F127 (1.0 g) was added into ethanol (10 ml) under stirring at 40 ^{o}C in a water bath. This solution was called solution A. Then, aluminum isopropoxide (3.0 g) was added to ethanol (10 ml) under stirring at 40 ^{o}C in a water bath. This solution was called solution B. Then, solution B was added to solution A under stirring. After 4 h of continuous stirring, the solution transformed into a translucent sol. Then, CCT (3.0 g) was added to the as-prepared sol. After impregnation, the solid-liquid mixture was filtered and dried in a vacuum oven at 50 ^{o}C for 12 h. The desired 3DOMM-Al_2O_3 support was obtained after calcining. For 3DOM and M-Al_2O_3, the synthesis procedure is similar to 3DOMM-Al_2O_3 except that no corresponding template is added in the synthesis process（F127 and PMMA, respectively).

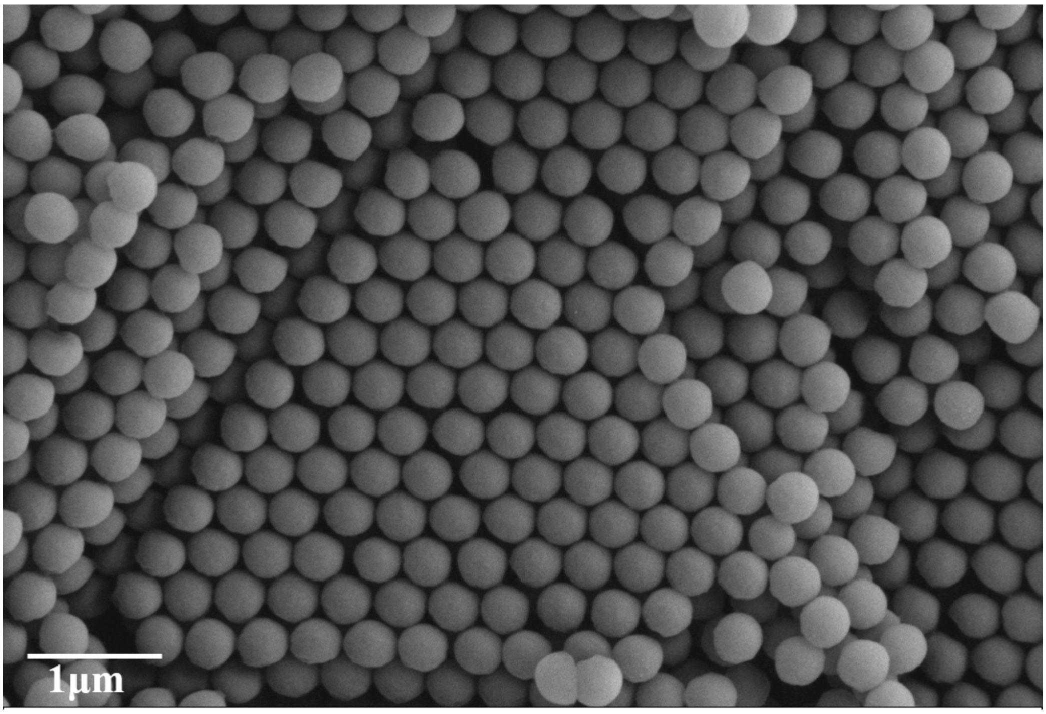

Figure S1. The morphology of 3D close-packed PMMA templates.

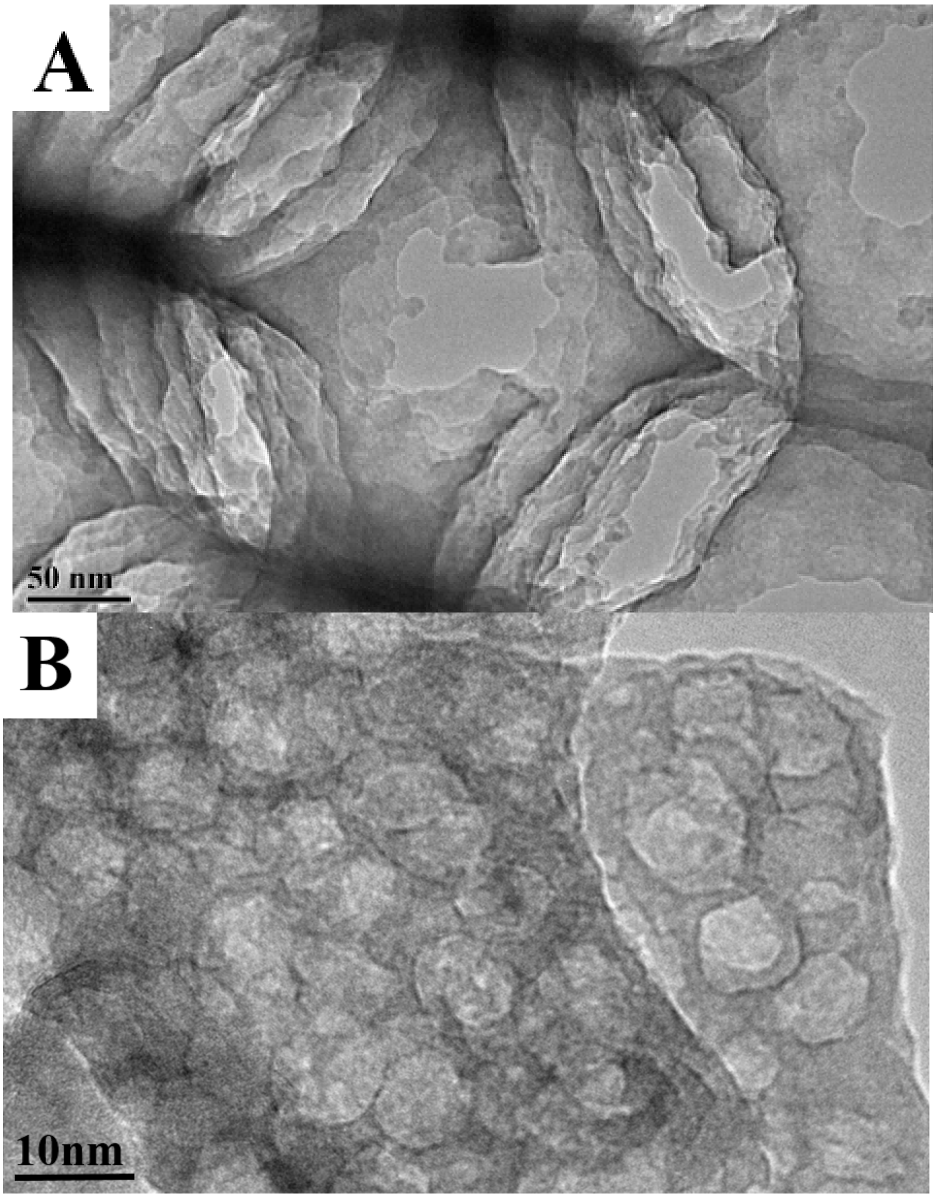

Figure S2. TEM images of 3DOM-Al_2O_3 (**A**) and M-Al_2O_3 (**B**).

Table S1. Performance comparison of recent Pt-based catalysts

Catalysts	Temperature °C	WHSV h^{-1}	Pt loading wt%	Conversion %	Selectivity %	Reference
PtSn/m-Al_2O_3	590	3	0.4	30	92	1
PtSn/c-Al_2O_3	600	3.2	0.5	36	89	2
Pt/Zn-ZSM5	590	3	0.5	41	93	3
Pt/OH-Al_2O_3	550	4	0.5	33	35	4
PtZn@S1	600	6.5	0.3	31	99	5
PtSn/3DOMM-Al_2O_3	600	3	0.5	51	91	This work

Reference

[1] Zhang, Y.; Zhou, Y.; Shi, J.; Zhou, S.; Sheng, X.; Zhang, Z.; Xiang, S. Comparative study of bimetallic Pt-Sn catalysts supported on different supports for propane dehydrogenation. Journal of Molecular Catalysis A: Chemical **2014**, 381, 138-147, doi: 10.1016/j.molcata.2013.10.007.

[2] Pham, H.N.; Sattler, J.J.H.B.; Weckhuysen, B.M.; Datye, A.K. Role of Sn in the Regeneration of Pt/γ-Al_2O_3 Light Alkane Dehydrogenation Catalysts. ACS Catal. 2016, 6, 2257-2264, doi: 10.1021/acscatal.5b02917.

[3] Zhang, Y.; Zhou, Y.; Huang, L.; Zhou, S.; Sheng, X.; Wang, Q.; Zhang, C. Structure and catalytic properties of the Zn-modified ZSM-5 supported platinum catalyst for propane dehydrogenation. Chem. Eng. J. **2015**, 270, 352-361, doi: 10.1016/j.cej.2015.01.008.

[4] Mironenko, R. M., Belskaya, O. B., Talsi, V. P., et al.; Effect of γ-Al_2O_3 hydrothermal treatment on the formation and properties of platinum sites in Pt/γ-Al_2O_3 catalysts. Applied Catalysis A: General 2014, 469, 472-482, doi.org/10.1016/j.apcata.2013.10.027.

[5] Wang, Y.; Hu, Z.; Lv, X.; Chen, L.; Yuan, Z. Ultrasmall PtZn bimetallic nanoclusters encapsulated in silicalite-1 zeolite with superior performance for propane dehydrogenation. J. Catal. **2020**, 385, 61-69, doi: 10.1016/j.jcat.2020.02.019.
